# Evaluation of a Community Reintegration Outpatient Program Service for Community-Dwelling Persons with Spinal Cord Injury

**DOI:** 10.1155/2014/989025

**Published:** 2014-12-09

**Authors:** Alana Zinman, Nicole Digout, Patricia Bain, Sylvia Haycock, Debbie Hébert, Sander L. Hitzig

**Affiliations:** ^1^Department of Occupational Science and Occupational Therapy, Faculty of Medicine, University of Toronto, Toronto, ON, Canada M5G 1V7; ^2^Lyndhurst Centre, Toronto Rehabilitation Institute, University Health Network, Toronto, ON, Canada M4G 3V9; ^3^Toronto Rehabilitation Institute, University Health Network, Toronto, ON, Canada M5G 2A2; ^4^Institute for Life Course and Aging, Faculty of Medicine, University of Toronto, 263 McCaul Street, Suite 328, Toronto, ON, Canada M5T 1W7

## Abstract

*Objective.* To evaluate the effectiveness of a community reintegration outpatient (CROP) service for promoting well-being and community participation following spinal cord injury (SCI). *Participants.* Community-dwelling adults (*N* = 14) with traumatic and nontraumatic SCI. *Interventions.* The CROP service is a 12-week (1 × week; 120 minutes) interprofessional closed therapeutic education service. *Main Outcome Measure(s).* Moorong Self-Efficacy Scale (MSES); Impact on Participation and Autonomy (IPA); Positive Affect and Negative Affect Scale (PANAS); Coping Inventory of Stressful Situations (CISS); World Health Organization Quality of Life (WHOQOL-BREF); semistructured qualitative interviews. *Methods.* Twenty-one participants were recruited from two subsequent CROP services, with only 14 persons completing all data assessments. Data were collected at baseline (week 0), at exit (week 12), and at a three-month follow-up. Semistructured interviews were conducted at exit. *Results.* Self-efficacy (MSES) and positive affect (PANAS) improved from baseline to exit (*P* < .05), but the changes were not maintained at follow-up. Qualitative analysis identified four major themes related to therapeutic benefits: (1) role of self; (2) knowledge acquisition; (3) skill application; and (4) group processes. *Conclusions.* Participation in a therapeutic education service has the potential to improve well-being in persons with SCI, but there is a need to identify strategies to maintain long-term gains.

## 1. Introduction

After sustaining a spinal cord injury (SCI), individuals must often cope with various physical, psychological, and social issues that occur as a result of their injuries [1]. The primary impairment of paralysis, along with the host of associated secondary health conditions (i.e., pain, depression, and bowel and bladder dysfunction) [2], causes significant burden to the individual and incurs substantial costs to the healthcare system. Recent data estimating the direct medical costs associated with traumatic SCI reported that the lifetime economic burden per individual ranges from $1.5 million for persons with incomplete paraplegia to $3.0 million for persons with complete tetraplegia [3]. Furthermore, SCI and associated conditions cause significant challenges for maintaining well-being in the community [4].

To mitigate the impact of the injury, people with SCI need to learn to adjust and accommodate to the resulting lifestyle changes [5]. The ability to adjust after SCI is often independent of the level and/or severity of injury; rather it is dependent on the coping strategies employed by the individual [6]. If an individual copes poorly with difficulties encountered during the reintegration process after SCI, then there is a greater likelihood of that person experiencing high levels of emotional distress, anxiety, and/or a depressive disorder [7, 8]. Thus, having a good repertoire of positive coping strategies can serve to manage common stressors associated with living with an SCI, which can contribute to better community participation and quality of life (QoL) [9].

There is a growing body of work on the use of self-management programs to help people with SCI address the challenges associated with living their injuries [10]. Self-management programs can serve to minimize the occurrence or impact of secondary health conditions by providing knowledge and skills related to risk and protective factors [11], while fostering appropriate coping mechanisms for a variety of life situations impacted by an SCI (e.g., employment, family relations, etc.) [10]. Although there are a variety of existing programs, such as the Stanford Chronic Disease Self-Management Program, it has been suggested that there is a need for more programs that are specifically tailored for people with SCI [12, 13]. For instance, programs need to provide information that is relevant to persons who have limited mobility or who are dependent on a wheelchair [13]. There is also a need for programs that can serve to enhance self-efficacy (*one's belief in his/her ability to succeed and manage challenging situations and accomplish goals* [14]) and psychosocial care after SCI [15] given that self-confidence or self-efficacy to manage one's SCI has been found to be suboptimal in this population [16].

The high costs associated with living with SCI and its impacts on physical and mental health indicate there is a need for more research on the effectiveness of self-management programs to promote well-being in persons with SCI. Thus, the primary objective of this study was to investigate the efficacy of the community reintegration outpatient (CROP) service for community-dwelling individuals with an SCI. The CROP is a closed therapeutic education service that imparts self-management strategies by offering education on various aspects of coping with an SCI (i.e., pain management, stress management, self-care, etc.), with the goal of enhancing community participation. It was hypothesized that persons who participated in the CROP service would demonstrate improvements in psychological, emotional, and social well-being. The findings from this study can serve to inform the development and implementation of SCI-specific self-management programs at other rehabilitation settings.

## 2. Methods

### 2.1. The Community Reintegration Outpatient (CROP) Service

The CROP service is a 12-week (1 × week) closed therapeutic education service, cofacilitated by an occupational therapist and social worker at a tertiary SCI rehabilitation hospital, where clients with SCI are provided with opportunities (1) to learn and understand the role of the “self” in the recovery process; (2) to share experiences and learn from one another; and (3) to identify and develop a visual roadmap for improving coping, well-being, and overall self-management skills while reintegrating back into the community.

The development of the CROP was initiated by an interprofessional group of clinicians providing SCI outpatient rehabilitation services in response to an identified gap in clinical practice related to community integration support after discharge from initial SCI rehabilitation. The clinicians noted that some clients with SCI were having difficulty reintegrating back into their communities and hypothesized that these challenges were partly attributable to low self-efficacy and limited opportunities for meaningful social participation (e.g., employment, leisure, etc.). To address these issues, the interprofessional team initiated the development of a specialized service to help persons with SCI successfully participate in their physical and psychosocial environment. Specifically, the service would provide a structured platform to enable people with SCI to reflect on their experiences living with the injury and acquire the necessary skills and knowledge to engage (or resume) in appropriate social roles, statuses, activities, and productive behaviours in “natural” community settings [17, 18]. With support from the organization, in-kind contributions (e.g., provision of space, clinical release time to develop the service, etc.) were provided to staff to establish and deliver a time-specific pilot project to promote better outcomes in the community for outpatients with SCI. The development of the CROP occurred through a series of systematic steps, which included (1) literature review; (2) development of a model of care; and (3) iterative service planning and quality improvement processes.

### 2.2. Literature Review

The first step towards developing the CROP was a review of the literature to identify barriers and facilitators influencing community participation following SCI. The review was focused on identifying physical, environmental, emotional, and social stressors associated with SCI. For instance, poorer health as a result of the injury [19], reduced employment opportunities [9, 20], limited social support and family role functioning [21, 22], limited access to recreational and leisure activities [23, 24], and a lack of accessible transportation [25, 26] were all noted to affect participation. There are also invisible and conceptual barriers that arise from the attitudes and beliefs of the individual with the SCI and from society as a whole. For example, a poor locus of control and the belief that a person is not capable of accomplishing the same things that he/she could do before injury can lead to him/her not being proactive in community life [1]. Successful integration back to the community following SCI requires new learning, problem solving, adaptation to lifestyle changes, and effective coping skills [9]. Along with the clinical experience of the interprofessional team, the review of the literature provided a working model on which topics needed to be addressed to support optimal community engagement after SCI.

### 2.3. Model of Care

With regard to client selection and delivery approach, existing clinical models and services were reviewed to identify best practices for the implementation of the CROP. In terms of finding appropriate clients interested and willing to attend the service, the clinicians working to develop the CROP service were providing SCI outpatient rehabilitation services within a therapeutic day program (TDP) to persons who were recently discharged from the inpatient rehab program. Although the TDP was initially identified as relevant source for recruitment to the first iteration of the CROP, there were drawbacks to this targeted recruitment strategy. Due to a limited number of patients in the TDP, staggered starting dates, and a high dropout rate due to other attended services' ending, it was decided to make the CROP service available to all patients accessing outpatient services.

With regard to service delivery, a group work approach was deemed as a clinically efficient way of providing the service. Group work processes help promote individual identity and enhance personal strengths, increase motivation and optimism, create sources of support, and provide an environment for constructive growth and problem solving [27]. In order to foster perceived control and self-discovery in participants, it was decided to use guided facilitation to manage the group process. Thus, a closed group model under the guidance of consistent facilitators was adopted for the planned therapeutic group service.

### 2.4. Service Planning and Quality Improvement

Service planning and quality improvement were an evolutionary process and served to further refine the CROP service. The use of outcome measures, group process facilitation skills, and the development of topic themes emerged through clinical observations and a formal client feedback process. A program logic model was used to provide a framework for developing the structure of the service. For instance, the group facilitation process was driven by the concept of the role of self, each patient's commitment to the group, and readiness for change and for self-management within the community.

Central to the development of the written content and take-home assignments were the barriers and success factors identified in the community participation literature review (described above). Each weekly session focused on active involvement in learning by incorporating education/lectures, reflections, and interactive discussions and activities. The group structure, process, and content were founded on multiple theoretical models including cognitive behavioural therapy [28], the Canadian Model of Occupational Performance [29], principles of adult learning [30, 31], group process theory [32], goal setting [33], and client-centered care.

The first iteration of the CROP was evaluated using the Reintegration to Normal Living Index (RNL) [34], a measure reflective of community participation, and through patient feedback surveys developed by the interprofessional team. Using feedback from the initial group of CROP service participants, the service developed a stronger emphasis on goal setting, self-efficacy, self-identity, and life roles. Unfortunately, the findings from the RNL indicated no improvements in community participation from baseline to program exit for CROP participants. Although the RNL is validated for use with SCI [35], the tool may not have been sensitive enough to detect changes over a relative short time since the version used employed a three-point numeric scale. Another potential limitation of the RNL is that it was not designed to assess changes in all of the targeted domains (e.g., coping, self-efficacy) deemed amenable by the CROP. Thus, it was determined that a more robust evaluation of the service was required, which included the use of more standardized measures that would be more appropriate for assessing the efficacy of the service (see “*instruments*” description below).

### 2.5. CROP Service

**Figure 1:**
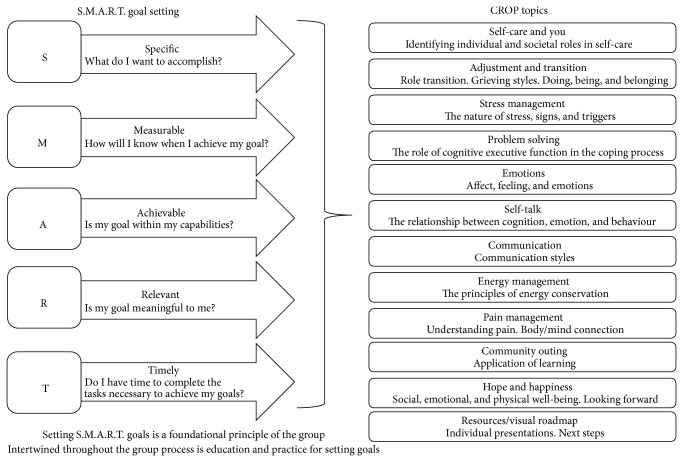
CROP S.M.A.R.T. goals and topics.

The revised CROP service and formal program evaluation framework was initiated in May 2011 (Session 1), with a subsequent session held in May 2012 (Session 2). The CROP service was held over a 12-week period, with each weekly session lasting approximately 120 minutes. The CROP was cofacilitated by a social worker and occupational therapist and was provided at no-cost to the participants. A different topic relevant to managing an SCI in the community was covered each week (see Figure 1). The selected topics for discussion were based on earlier feedback from the participants of the initial CROP session (described above) and were reflective of the issues noted in the SCI literature and the clinical problems being reported by the patients attending the outpatient services at the rehabilitation centre (e.g., pain, stress, etc.).

**Figure 2:**
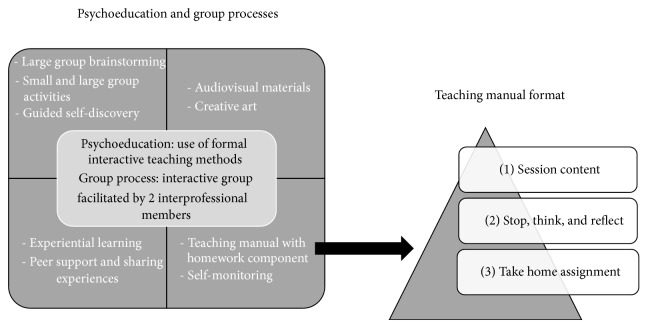
CROP processes and materials.

A key feature for facilitating personal growth and group discussion was a goal setting process. At the initial CROP session (week 1), participants established a specific goal they wanted to accomplish over the 12-week service (see Figure 1), which was formulated by using a S.M.A.R.T. approach (specific, measureable, attainable, realistic, and time-sensitive). Strategies for accomplishing the selected goals of the participants were discussed by the group on a rotating basis, with each member having an opportunity to discuss their progress and to receive feedback from the group at regular intervals on how to overcome any challenges or obstacles they were encountering. To facilitate the weekly discussions and to support the goal attainment process, a teaching manual designed by the social worker and occupational therapist who implemented the service was provided to each participant on the different topics (see Figure 2). The teaching manual contained information sheets about a variety of topics, which included self-care, stress management, energy conservation, emotional adjustment, and coping strategies (see Figure 1). The components of the manual were informed by the literature review and existing clinical materials and were subsequently vetted by several clinical staff members of the SCI outpatient service team. Each session also assigned weekly homework tasks, which consisted of simple and more in-depth reflection exercises (see Figure 2). An example of a simple homework task was to write down and complete a “Do One Thing” cue card, which encouraged participants to undertake an action step related to the topic of the week (e.g., making a phone call to a friend). An example of an in-depth reflection assignment was a “Stop, Think, and Reflect” question related to each session's content. For example, the “Session 2—Self Care” questions included “What are the top 5 things I value in life? Is maintaining good health and well-being on this list? If not, why?” and “What do I focus the majority of my time and energy on?” Although regularly assigned, completion of the homework was not mandatory. Different visual and learning aids were also used throughout the CROP services (see Figure 2). In order to provide a “real-world” opportunity to implement skills and knowledge gained from the CROP, one session took place in a community setting towards the end of the service. Overall the selected topics for discussion, the goal setting exercises, minihomework assignments, learning aids, and community outing were all designed to increase participant motivation to acquire skills and knowledge for community living from the interprofessional team while facilitating opportunities for the group to share their experiences of managing their SCI with one another. It should be noted that the CROP services were implemented on a trial basis and not part of standard clinical care at the SCI rehabilitation centre.

### 2.6. Participants

A convenience sample of twenty-one adults (10 men; 11 women) with traumatic and nontraumatic SCI was recruited from two subsequent CROP services (Session 1: May 2011 to July 2011; and Session 2: May 2012–July 2012). Inclusion criteria were community-dwelling adults (18 years or older) with traumatic or nontraumatic SCI, who were less than 3 years after injury/onset, who were fluent in English, and could attend the 12 weekly sessions of the CROP service. The exclusion criteria were persons who were not medically stable, who were not fluent in English (to the extent it would create a barrier for participation), or who had a cognitive impairment.

### 2.7. Instruments

#### 2.7.1. World Health Organization Quality of Life (WHOQOL-BREF)

The WHOQOL-BREF is a measure of QoL, grouped into four domains: physical capacity, psychological well-being, social relationships, and environment [36]. Higher scores on each subscale indicate better QoL. It has demonstrated excellent responsiveness with SCI and for program evaluation in rehabilitation [37]. Cronbach's alpha coefficients for the physical, psychological, social, and environment factors were computed to be 0.82, 0.82, 0.74, and 0.80, respectively [37].

#### 2.7.2. Coping Inventory for Stressful Situations (CISS)

The CISS measures three main coping strategies that people may use in stressful situations: task-oriented, emotion-oriented, and avoidance-oriented approach (distraction and social diversion) [38]. Studies evaluating the tool have concluded it has good predictive validity [39]. Cronbach alpha coefficients for the Problem, Emotion, and Avoidance scales for the CISS were found to be 0.91, 0.89, and 0.84, respectively. Test-retest reliability ranges from 0.76 to 0.90 [38].

#### 2.7.3. Positive Affect and Negative Affect Schedule (PANAS)

The PANAS measures positive and negative constructs as both states and traits [40]. A positive affect and negative affect subscale are calculated. The PANAS has demonstrated reliability among a sample of patients who have received inpatient medical rehabilitation, with a test-retest ICC of 0.79 for positive affect and 0.93 for negative affect [40]. In the general adult population, Cronbach's alpha has been demonstrated to be 0.89 and 0.85 for positive affect and negative affect, respectively [41, 42].

#### 2.7.4. Moorong Self-Efficacy Scale (MSES)

The MSES is a 16-item SCI self-efficacy scale, scored on a Likert scale from 1 to 7 [43]. Higher scores indicate higher levels of self-efficacy. The scale has good internal consistency and a test-retest reliability of 0.74 [44]. Significant correlations with self-concept measures, emotional distress scales, and functional independence measures demonstrate the validity of the MSES [44].

#### 2.7.5. Impact on Participation and Autonomy (IPA)

The IPA quantifies limitations in participation and autonomy via five subscales: autonomy indoors, family role, autonomy outdoors, social life and relationships, and work and education [45]. Higher scores represent poorer participation and autonomy. Test-retest reliability from a cross-disability sample, including SCI, ranges from 0.56 to 0.90 [46]. In one SCI study, the IPA had high internal consistency and ICCs, with all values greater than 0.70 [47]. In the same study [47], the minimal detectable change by IPA domain was found to be 0.70 for autonomy indoors, 1.18 for autonomy outdoors, 0.83 for family life, 0.76 for social life and relationships, and 0.86 for work and education.

#### 2.7.6. Qualitative Interviews

Semistructured interviews with participants were conducted at the end of the CROP service (week 12) to gain their insights about their participation and to gain feedback on how the service could be improved. The interview guide is described in the Appendix.

### 2.8. Procedure

A nonrandomized single arm study design was employed. Survey data were collected prior to participation (baseline), at completion (exit), and at 3 months after intervention (follow-up) by members of the research team. Participants also underwent semistructured interviews, which asked about their perceptions of the CROP service.

**Figure 3:**
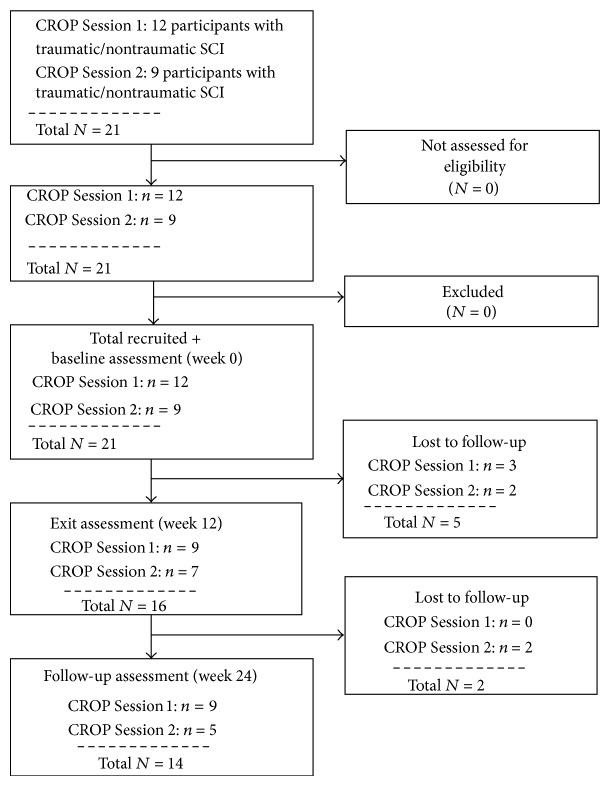
CROP service participant recruitment and assessment flow diagram.

As noted, participants were recruited from two CROP services (Sessions 1 and 2). Twelve participants were recruited at Session 1 and 9 participants were recruited at Session 2. Sixteen participants completed baseline and exit assessments; 14 of them completed baseline, exit, and follow-up assessments (see Figure 3 with flow diagram). Twelve participants took part in the semistructured interview at exit. Each interview lasted approximately 30 minutes. All interviews were audio-taped and transcribed for data analysis.

This study was approved by the Research Ethics Board of the Toronto Rehabilitation Institute and the University of Toronto, and we certify that all applicable institutional and governmental regulations concerning the ethical use of human volunteers were followed.

### 2.9. Analysis

Descriptive statistics and frequencies were used to describe the sample and scores on the outcome measures. For participants who only completed the baseline and exit assessments (*n* = 16), the data met the assumptions for normality, and thus paired *t*-tests were conducted. Effect sizes were calculated following the procedures described by Lakens [48]. This included providing common language (CL) effect sizes, which converts effect sizes into percentages, and

“expresses the probability that a randomly sampled person from one group will have a higher observed measurement than a randomly sampled person from the other group (for between-designs) or (for within-designs) the probability that an individual has a higher value on one measurement than the other.” [48, page 4].

For those who completed baseline, exit, and follow-up assessments (*n* = 14), Friedman tests were used to analyze the data given the small sample size and because not all of the data met the assumptions of normality. Post hoc comparisons were conducted using Wilcoxon *t*-tests with a bonferroni correction. Effect sizes for these analyses were calculated using the formula *r* = *Z*/√*n*.

For the qualitative data, an inductive content analysis was conducted [49–52]. This process involved using open-coding and creating categories that emerged from the participant transcripts [52]. Two investigators independently coded each transcript and regularly met to corroborate their findings in order to form a decision of what aspects of the interview belonged under the same category. Points of disagreement were resolved through discussion and documented through an audit trail. The technique of “code-recode” was conducted to verify content validity, and major themes and associated subthemes were identified. Investigator triangulation was used at each stage of the analysis process to ensure trustworthiness of the data [51]. This included involving a third investigator who confirmed the subsequent coding frameworks served to resolve points of disagreement between the two main coders. The end-goal of the data analysis process was to achieve saturation, in which no new information emerges from the transcripts [50].

## 3. Results

### 3.1. Quantitative Analysis

Table 1 presents the sociodemographic and injury profiles of persons who completed baseline and exit (*n* = 16) and the sample characteristics of those who completed the baseline, exit, and follow-up assessments (*n* = 14). It should be noted that no differences emerged between persons from CROP Session 1 and CROP Session 2 in terms of sociodemographics (age, gender) or impairment (etiology, level of injury, severity of injury, and months after onset).

### 3.2. Changes between CROP Baseline and Exit Scores

For persons who completed both baseline and exit assessments (*n* = 16; Table 2), there was a significant increase in self-efficacy from baseline (*M* = 68.6, SD = 15.6) to exit (*M* = 77.6, SD = 16.1), *t*(15) = 3.90, *P* = 0.001, 95% CI [4.05, 13.82], and Hedges's grm = 0.55. The common language (CL) effect size indicates that after controlling for individual differences, the likelihood that a person would score higher on self-efficacy at exit than at baseline is 83%. With regard to positive affect, there was an increase in scores from baseline (*M* = 30.75, SD = 7.4) to exit (*M* = 38.3, SD = 8.0), *t*(15) = 3.97, *P* = 0.001, 95% CI [3.48, 11.53], and Hedges's grm = 0.95. The CL effect size indicates that after controlling for individual differences, the likelihood that a person would score higher on positive affect at exit than at baseline is 84%. Conversely, scores on negative affect decreased from baseline (*M* = 26.19, SD = 10.6) to exit (*M* = 22.1, SD = 8.3), *t*(15) = −2.17, *P* = 0.047, 95% CI [−8.06, −0.06], and Hedges's grm = 0.40. The CL effect size indicates that after controlling for individual differences, the likelihood that a person would score lower on negative affect at exit than at baseline is 71%.

With regard to task-oriented coping style as measured by the CISS, the mean score at exit (*M* = 64.3, SD = 8.1) was significantly higher than the mean score at baseline (*M* = 56.1, SD = 7.1), *t*(15) = 3.05, *P* = 0.008, 95% CI [2.45, 13.80], and Hedges's grm = 1.04. The CL effect size indicates that after controlling for individual differences, the likelihood that a person would score higher on CISS task-orientation at exit than on baseline is 77%. In terms of CISS avoidance-oriented coping, the mean score at exit (*M* = 48.6, SD = 11.0) was significantly higher than the mean score at baseline (*M* = 42.2, SD = 10.7), *t*(15) = 2.61, *P* = 0.020, 95% CI [1.17, 11.70], and Hedges's grm = 0.58. The CL size indicates that after controlling for individual differences, the likelihood that a person would score higher on CISS avoidance-oriented coping at exit than at baseline is 74%. Similarly, scores on CISS social diversion were significantly higher at exit (*M* = 16.9, SD = 5.2) than at baseline (*M* = 14.6, SD = 4.4), *t*(15) = 2.74, *P* = 0.015, 95% CI [0.511, 4.11], and Hedges's grm = 0.46. The CL effect size indicates that after controlling for individual differences, the likelihood that a person would score higher on CISS social diversion at exit than at baseline is 75%.

In terms of QoL, there was an increase in psychological QoL (WHOQOL-BREF) from baseline (*M* = 18.38, SD = 3.6) to exit (*M* = 19.75, SD = 3.8), *t*(15) = 2.39, *P* = 0.031, 95% CI [0.15, 2.60], and Hedges's grm = 0.36. The CL effect size indicates that after controlling for individual differences, the likelihood that a person would score higher on psychological QoL at exit than at baseline is 72%. With regard to community participation, there was a significant decrease in the perceived barriers to autonomy in the outdoors (IPA) from baseline to exit, with scores decreasing from (*M* = 11.4, SD = 5.2) to (*M* = 9.3, SD = 4.5), *t*(15) = −2.75, *P* = 0.015, 95% CI [−3.89, −0.49], and Hedges's grm = 0.43. The CL effect size indicates that after controlling for individual differences, the likelihood that a person would have a better score on perceived outdoor autonomy at exit than at baseline is 75%.

### 3.3. Changes across CROP Baseline, Exit, and Follow-Up Scores

For persons who completed all the assessments (*n* = 14; Table 3), Friedman test indicated a significant difference for self-efficacy scores across time (MSES; *ϰ* ^2^ = 7.259, *P* = 0.027). Post hoc comparisons revealed that self-efficacy scores at exit (median = 77.5) were significantly higher (*P* = 0.003) than baseline scores (median = 68.5) and that the increase was moderate in size (*r* = 0.56). However, no differences emerged between baseline and follow-up (median = 77.5) scores nor between exit and follow-up self-efficacy scores. Positive affect improved over time (PANAS; *ϰ* ^2^ = 7.259, *P* = 0.027), with the difference (*P* = 0.009) only emerging between baseline (median = 31.5) and exit (median = 39.0) scores, and the size of this increase was moderate (*r* = 0.50). Although there was a significant difference detected for physical QoL (WHOQOL-BREF; *ϰ* ^2^ = 7.840, *P* = 0.020), post hoc comparisons negated this effect.

### 3.4. Qualitative Analysis

Four major themes (Table 4) related to therapeutic benefits emerged from the semistructured interviews: (1) role of self, (2) knowledge acquisition, (3) skill application, and (4) group processes. In addition, satisfaction with the CROP service was identified as a theme and subcategorized into positive and negative perceptions of the service, as well as a subcategory describing suggestions for CROP service improvement.

#### 3.4.1. Role of Self

Participants spoke about finding themselves and their “post-SCI identity” through their participation in the program. The therapeutic benefits gained with regard to “role of self” included improved self-esteem, self-confidence, and a better understanding of their limitations associated with SCI.

The need to be assertive and advocate for necessary care to achieve important goals was also expressed by the sample. Some participants spoke of the importance of self-advocacy, specifically as it related to communicating their needs and limitations to caregivers, friends, and/or family. Participants expressed that their participation in the service enabled them to better assert themselves, which was related to their gains in self-confidence and a better understanding of their needs:

“Suddenly I'll vocalize limitations, so they're like, are you wimping out on us… cause before I was like strong and I'm still strong, but now… I want a more balanced life… I respect my body a lot more.” (ID number 115)

“… it's also helped me deal with, like being more assertive, that was always an issue for me… like being able to say what I want to say instead of being quiet.” (ID number 1209)

They were also better able to communicate this lifestyle change to their family:

“… I've got the language.” (ID number 115)

Timing of the service was also critical for some participants who recently were transitioned from inpatient to outpatient rehabilitation and were readjusting to community living. Gaining insight was frequently expressed and was a salient theme that emerged across interviews. For instance, participants gained not only insight into the limitations and challenges associated with an SCI but also the ability to accept the limitations and move forward with a positive outlook:

“It was a combination of learning about myself and you know how my situation relates to other people's situations.” (ID number 119)

“And I think it was [social worker] who said that sometime we're not even aware of emotions that are really kind of bogging us down, and I wasn't, and I think that as hard as it was at times facing those emotions, like the sadness and the loss that I feel… actually having faced them… the thing is that I feel lighter… like I can see myself opening up more in terms of accepting my limitations, I'm so much better.” (ID number 115)

“I mean you can always throw your towel in and surrender… or you can you know just smarten up and say-okay yes you know come to the realization of your present circumstances and deal with it.” (ID number 120)

“It (the CROP program) just changed my whole outlook on life and it's made a lot of positive changes.” (ID number 1203)

“… it helped me kind of realize I can, you know, do stuff on my own and, you know, everything would be okay, and how to deal with different things, emotions and all that kind of stuff that you're going through.” (ID number 1209)

Most importantly, the CROP service instilled hope in many of the participants, and they expressed that the program opened up possibilities for the future:

“It has widened my horizon as to the possibilities. Things could get better and you know… you know other things will come in.” (ID number 120)

“Psychologically, intellectually it's really… It changed my life… In the way that I think… and there is still some light.” (ID number 121)

“And in going through this program slowly got me to turn around and look more at what I still could do and why I should feel lucky rather than depressed.” (ID number 1203)

“It just helped to show us that there's still a hell of a lot that we can be thankful for.” (ID number 1203)

#### 3.4.2. Knowledge Acquisition

Participants spoke about how they acquired knowledge by participating in the program. Specifically, they described how they gained knowledge related to their SCI, which included self-management strategies. The knowledge and strategies were derived from the educational materials and from their “interactions with the group.” Many of the participants were first interested in participating in the CROP service to acquire skills for community participation:

“So life after SCI, if there's anymore… Like anything I can gain to help me integrate back into society.” (ID number 116)

The CROP service allowed them to acquire specific skill sets and tools to assist them in the community:

“I mean just like learning about the different issues and how not to just live with them, how to live well with them.” (ID number 115)

“It was really good… especially the resources that they can provide you… like readings and different materials… cause now you have all this information so, you know, if I need to look at something if I'm dealing with stress or something emotional, sometimes I just go and kind of look back at what we did and it's like oh yeah, I can deal with it, things in a different or better way.” (ID number 1209)

#### 3.4.3. Skill Application

Participants spoke about the opportunity the service provided them on being able to implement the skills gained in the community setting. Comments related to this were specifically related to the community outing undertaken by the group, which was done with support by the clinical facilitators. The community outing challenged them to use the skills that were taught throughout the group, such as energy conservation:

“The outing was really useful… it was a good application of what we learned and talked about for all those weeks. Because like before you heard pace yourself, take breaks when you need to… then you go there and you do the exact opposite, you know? Until it starts taking its toll and then you realize-oh no I'm supposed to stop and rest. You know and you sort of reflect back at all those things were taught.” (ID number 119)

Participants also spoke about how they applied the specific skills they learned (i.e., stress management) to their everyday lives:

“For me it was just coping you know… you know the caregivers… and the course taught me how to take control, you know of my care and with the realization that nobody's gonna look after you like you.” (ID number 116)

Many participants also expressed the desire to participate more actively in their community post-CROP service by setting short- and long-term goals, with a number of them relating specifically to work and leisure activities:

“I've been doing the computer classes at the spinal cord resource centre… maybe I might end up being able to go back to work. But when I started out I had a grade 8 education, drove (a) truck all my life. I can't go back to that… I had nothing to offer anybody. Now I'm getting these courses… and we'll see what happens.” (ID number 1203)

“One of the sessions was… make a goal and do it… like trying, you know, different new sports. Like I'm going into a marathon, which I would have never done before… I'm just gonna do it. And I don't think I would have done that before without some of the issues that we discussed and having that confidence…” (ID number 1209)

#### 3.4.4. Group Processes

Participants spoke about the group dynamics and supportive environment facilitating their learning and experiences in the CROP service. They were able to share their own knowledge and experiences of SCI and also learn from the experiences of others. Involvement in a group provided participants with an opportunity to reflect on how their condition was similar or different from other patients:

“You know cause I felt like as someone who was once an able bodied person and now facing this new challenge of mobility and… I just wanted to get other people's take on it and see if I can benefit and if I can share any of my experiences with them also.” (ID number 119)

The same participant also mentioned that

“The group dynamic that I participated in was just phenomenal in the sense that everyone… participated, everyone gave some input… Like it was a real sharing.” (ID number 119)

Group dynamics appeared to be an important factor of the group since they were able to relate to one another and discuss their struggles managing their SCI.

#### 3.4.5. Program Satisfaction

The group was highly satisfied with the CROP service, particularly among the following areas: (1) supportive environment/facilitators, (2) format/topics, (3) resources, and (4) community outing. However, a majority of the group members felt that more time was needed for each session. They also felt that the program could have been longer overall (e.g., more sessions). Many of the participants spoke about how a follow-up service or additional resources after CROP completion would be beneficial for helping them to maintain their perceived gains in well-being.

## 4. Discussion

The aim of this project was to evaluate a therapeutic education service, namely, the CROP service, for improving well-being in community-dwelling persons with SCI. The findings indicate that there were a number of therapeutic benefits at the end of the service, with the gains in self-efficacy and positive affect having the most robust effect. However, the changes in these domains were not maintained over time (3 months later). Similarly, the patterns of scores for the other targeted domains, albeit nonsignificant, were in the expected direction but also returned towards baseline values at the 3-month follow-up. It is important to note that managing an SCI is a lifelong process due to the many secondary conditions that can occur [53] and not uncommon for someone to experience three to eight health conditions at any given time [2, 22]. Experiencing even one moderate or severe health condition (e.g., pressure ulcer) can have a significant impact on physical, psychological, and social well-being [4, 6, 8, 11]. Hence, the lack of significant findings at the follow-up assessment might be attributable to some of our participants having a “flare-up” of health conditions that impacted their well-being across a number of areas. Overall, our hypotheses were only partially confirmed, but the moderate and reliable effect sizes related to improved mood and self-efficacy provide important evidence for the clinical utility of the CROP service.

The qualitative analysis revealed that participants experienced therapeutic gains and were highly satisfied with the service, which provides additional evidence on the perceived value of the CROP service. Many of the participants felt they gained relevant knowledge and coping skills for community participation and valued the opportunity for sharing their insights with peers. The comments provided by the participants also suggest that the domains the outcome measures assessed were appropriate (e.g., gains in self-confidence). The lack of significant findings on the standardized outcome measures may have been due to the need for a follow-up service or additional resources after CROP completion to sustain the positive effects of the program over time. This issue was highlighted by the participants, who felt that having an additional or “booster” session following the service would be helpful. Although the service was held over an intense three-month period (12 weeks × 1 session per week for 120 minutes each session), the need for more time was a salient theme, which suggests either the weekly sessions could have been extended or perhaps the intensity of the program could be delivered in wider intervals (e.g., every two weeks). There is some evidence on the effectiveness of self-management programs after SCI that are implemented across wider time periods (e.g., bimonthly) and that have longer sessions (e.g., half-day) [54].

The findings regarding self-efficacy are particularly noteworthy since it is a key construct associated with positive outcomes after SCI [16, 54]. Several other self-management programs also strive to improve self-efficacy in their clients [13, 54, 55]. For instance, in “Project Shake-It-Up,” which is a health promotion and capacity building program for people with SCI, multiple sclerosis, and related neurological impairments, it was found that self-efficacy increased in participants compared to nonparticipants and that these gains were maintained over a 12-month period [54]. The maintained increases in self-efficacy might be attributable to the program incorporating leisure-based activities and taking place entirely in the community. For instance, participants were provided with opportunities to engage in a variety of indoor and outdoor physical recreational activities (e.g., strength training, sailing, sea kayaking, hand cycling, etc.) each afternoon of the program, while the morning seminar sessions took place in different community-based settings (e.g., libraries, university campuses, state parks, etc.). As such, the opportunity to “learn” in the community and to engage in “physical/recreational” activities may have provided an additional boost towards elevating and maintaining self-efficacy in the “Project Shake-It-Up” participants. Engagement in physically active recreational activities has been shown to elevate both mood and self-efficacy in people with SCI [56]. Although the CROP service did provide a community outing, there might be a need for more opportunities for the group to practice the skills and knowledge learned in a variety of community settings.

Based on the findings from other self-management programs [13, 54, 55], along with the demonstrated increases in mood and self-efficacy in the present sample, it appears that the tools, resources, and support provided by the CROP service provided participants with the perception that they have the skills to manage challenges and achieve their goals for community living. Participants expressed that the group processes within a supportive environment facilitated their learning and promoted therapeutic gains. Engaging in group sessions may have contributed to a significant increase in self-efficacy since social comparison is an important mechanism for self-efficacy [13].

The themes that emerged from the interviews are closely related to the phenomenon of “posttraumatic growth.” This describes individuals who have experienced a traumatic event and have come to view the event as an avenue for personal development and growth [57]. This perception tends to lead to positive outcomes, such as (1) improved interpersonal relationships, (2) positive change in the perception of the self, and (3) an emerging or developing philosophy of life [57]. Themes such as gaining insight, group dynamics, and self-development suggest that participants may have been describing their experience of posttraumatic growth. Although further work is required to explore this construct, participation in the CROP service may serve to foster posttraumatic growth for this population.

The evaluation of the CROP service was done to provide information on its impact for helping people with SCI maintain health and well-being in the community. At this time, the CROP service is only being provided on a pilot basis, and data supporting its efficacy will serve to determine its value for including it as part of standard clinical care. The evaluation also provided important information related to decision making on the CROP service implementation since there is a need to further refine strategies on how initial gains can be maintained over time. Relatedly, program evaluation should be an on-going process to ensure that clinical programs are effectively meeting the needs of their clients. The present evaluation was framed within a research perspective but future evaluations will work to refine the selection and use of outcome measures in order to provide information that is clinically meaningful to both clinicians and patients to aid in evaluating the CROP service's efficacy at the individual level [58]. Doing so may better provide insight to what processes promote immediate gains after service in the participant and what additional supports they can access to maintain their long-term gains.

### 4.1. Study Limitations

A limitation of the current study is the small size of our sample, which may have accounted for our lack of significant findings. Further work using a larger sample size may conclusively demonstrate the effectiveness of the CROP service. However, the findings from the qualitative component achieved saturation, which indicates a number of positive outcomes associated with participating in the service. A second limitation is that participant interviews were conducted at completion of the service. A follow-up interview at the three-month follow-up may have provided additional insight on why the gains in self-efficacy and positive affect were not maintained over time. It is also possible that the group did undergo some actual changes in the ability to cope with living with an SCI in the community, but our long-term follow-up surveys may not have been suited or sensitive enough to capture these changes. The use of a “waiting list” control group may have also helped to demonstrate if the changes in scores were directly attributable to participation in the CROP. Another limitation was the inability to follow-up with our entire sample on all of the planned assessments. Only 16 of the initial 21 persons completed the baseline and exit assessments and only 14 completed all three assessments. The loss of participants across assessment intervals may have affected our outcomes.

## 5. Conclusion

There is a need for effective interventions for improving community participation and QoL after SCI, and the CROP service is a promising intervention for helping people with SCI to achieve this goal. Further work is required to help participants maintain the long-term therapeutic gains in the community but is an important service that provides skills and knowledge to people with SCI on how to better manage the emotional, environmental, and social stressors that challenge community participation.

## Clinical Messages

- Sustaining a spinal cord injury (SCI) creates a number of challenges for maintaining health and well-being in the community. Self-management programs, such as the community reintegration outpatient (CROP) service, are promising for helping people to offset regular stressors associated with SCI.
- Self-management programs using a group approach might contribute to gains in self-efficacy and positive affect, but follow-up sessions or additional resources might be required to sustain therapeutic gains over time.

## Figures and Tables

**Table 1:** Sample characteristics.

	Baseline *n* = 21	Baseline and exit *n* = 16	Baseline, exit, and follow-up *n* = 14
Gender (%)			
Male	10 (47.6%)	8 (50.0%)	8 (57.1%)
Female	11 (52.4%)	8 (50.0%)	6 (42.9%)
Age (M; SD)	46.0 (11.4)	46.3 (10.1)	46.6 (10.1)
Months after injury (M; SD)	44.6 (64.5)	52.2 (70.4)	41.4 (61.8)
Education (%)			
Less than postsecondary	3 (14.3%)	3 (18.8%)	3 (21.4%)
Postsecondary	18 (85.7%)	13 (81.3%)	11 (78.6%)
Employment (%)			
Employed	3 (14.0%)	2 (12.5%)	2 (14%)
Unemployed/LTD	18 (86.0%)	14 (87.5%)	12 (86%)
Trauma (%)			
Traumatic	14 (66.7%)	11 (68.8%)	10 (71.4%)
Nontraumatic	7 (33.3%)	5 (31.3%)	4 (28.6%)
Level (%)			
Paraplegia	7 (33.3%)	5 (31.3%)	4 (28.6%)
Tetraplegia	12 (57.1%)	9 (56.3%)	9 (64.3%)
N/A	2 (9.5%)	2 (12.5%)	1 (7.1%)
Severity (%)			
Complete	4 (19.0%)	2 (12.5%)	2 (14.3%)
Incomplete	15 (71.4%)	12 (75.0%)	11 (78.6%)
N/A	2 (9.5%)	2 (12.5%)	1 (7.1%)

M: mean; SD: standard deviation; %: percent.

**Table 2:** CROP scores: baseline and exit (*n* = 16).

Scale	Baseline M (SD)	Exit M (SD)	$\overset{-}{X_{D}}$ (SD)	95% CI	*P*
CISS: task-oriented	56.1 (7.1)	64.3 (8.1)	8.1 (10.6)	[2.5, 13.8]	0.008
CISS: emotion-oriented	48.1 (11.2)	42.8 (11.8)	−5.3 (10.2)	[−10.7, 0.2]	0.057
CISS: avoidance	42.2 (10.7)	48.6 (11.0)	6.4 (9.9)	[1.2, 11.7]	0.020
CISS: distraction	20.4 (5.9)	22.6 (5.5)	2.3 (6.4)	[−1.2, 5.7]	0.180
CISS: social diversion	14.6 (4.4)	16.9 (5.2)	2.3 (3.4)	[0.5, 4.1]	0.015
IPA: autonomy indoors	7.7 (7.9)	6.9 (6.5)	−0.8 (4.2)	[−3.0, 1.5]	0.483
IPA: autonomy outdoors	11.4 (5.2)	9.3 (4.5)	−2.2 (3.2)	[−3.9, −0.5]	0.015
IPA: family role	14.7 (7.9)	12.1 (6.3)	−2.6 (5.5)	[−5.5, 0.4]	0.084
IPA: social life	7.1 (5.8)	8.4 (5.6)	1.3 (5.0)	[−1.3, 4.0]	0.307
IPA: work and education^a^	7.9 (6.1)	5.9 (4.7)	−2.0 (6.0)	[−5.8, 1.8]	0.275
MSES	68.6 (15.6)	77.6 (16.1)	8.9 (9.2)	[4.1, 13.8]	0.001
PANAS PA	30.8 (7.4)	38.3 (8.0)	7.5 (7.6)	[3.5, 11.5]	0.001
PANAS NA	26.2 (10.6)	22.3 (8.3)	−4.1 (7.5)	[−8.1, −0.1]	0.047
WHOQOL: physical	20.7 (2.8)	20.9 (3.6)	0.3 (2.5)	[−1.1, 1.6]	0.697
WHOQOL: psychological	18.4 (3.6)	20.0 (3.8)	1.4 (2.3)	[0.1, 2.6]	0.031
WHOQOL: social	9.4 (2.6)	9.6 (2.5)	0.1 (2.0)	[−1.0, 1.2]	0.809
WHOQOL: environment	26.9 (5.2)	28.2 (5.6)	1.2 (4.8)	[−1.3, 3.8]	0.315

CISS: Coping Inventory for Stressful Situations; IPA: Impact on Participation and Autonomy; MSES: Moorong Self-Efficacy Scale; PANAS PA: Positive and Negative Affect Scale; PA: positive affect; NA: negative affect; WHOQOL-BREF: World Health Organization Quality of Life.

${}^{\text{a}}n=13;\;\overset{-}{X_{D}}$: mean difference; 95% CI: 95% confidence interval of the mean difference; SD: standard deviation; *P*: *P* value.

**Table 3:** CROP baseline, exit, and follow-up scores (*n* = 14).

Scale	Baseline M (IQR)	Exit M (IQR)	Follow-up M (IQR)	*P*
CISS: task-oriented	57.0 (13.0)	66.0 (9.0)	60.5 (17.0)	0.223
CISS: emotion-oriented	50.0 (18.0)	45.5 (20.0)	44.0 (22.0)	0.166
CISS: avoidance	41.5 (14.0)	48.0 (21.0)	43.0 (11.0)	0.089
CISS: distraction	22.0 (9.0)	22.5 (9.0)	16.5 (12.0)	0.102
CISS: social diversion	14.5 (6.0)	16.0 (10.0)	17.0 (6.0)	0.074
IPA: autonomy indoors	6.5 (11.0)	7.0 (11.0)	4.0 (11.0)	0.247
IPA: autonomy outdoors	12.5 (6.0)	10.0 (7.0)	12.0 (9.0)	0.199
IPA: family role	17.0 (13.0)	12.5 (10.0)	9.5 (14.0)	0.083
IPA: social life	7.9 (10.0)	7.5 (10.0)	7.5 (7.0)	0.945
IPA: work and educational^a^	7.0 (11.0)	4.0 (6.0)	4.0 (12.0)	0.559
MSES	68.5 (19.0)	77.5 (26.0)	77.0 (17.0)	0.027
PANAS PA	31.5 (15.0)	39.0 (11.0)	33.5 (14.0)	0.027
PANAS NA	26.0 (21.0)	19.0 (10.0)	24.0 (16.0)	0.584
WHOQOL: physical	21.0 (3.0)	21.0 (6.0)	22.5 (5.0)	0.020
WHOQOL: psychological	18.5 (6.0)	19.5 (7.0)	19.0 (6.0)	0.247
WHOQOL: social	10.0 (6.0)	10.0 (7.0)	9.0 (5.0)	0.472
WHOQOL: environment	25.0 (6.0)	29.0 (9.0)	27.5 (10.0)	0.410

CISS: Coping Inventory for Stressful Situations; IPA: Impact on Participation and Autonomy; MSES: Moorong Self-Efficacy Scale; PANAS PA: Positive and Negative Affect Scale; PA: positive affect; NA: negative affect; WHOQOL-BREF: World Health Organization Quality of Life.

^a^ *n* = 12; M: median; IQR: interquartile range; *P* = *P* value.

**Table 4:** Themes and subthemes related to CROP service participation.

Theme	Role of self	Knowledge acquisition	Skill application	Group processes
Subtheme	Gaining insight	Learning about SCI	Specific skill set	Group dynamics
	Assertiveness		Community participation	Share knowledge
	Self-confidence	Skill acquisition		Learn from others
	Self-development	Tools		
	Timing of service	Topics		Supportive environment

## Appendix

The purpose of this interview is to get an understanding of your experiences from participating in the CROP service.

Question  1: what were your expectations of the CROP service?
Question  2: what aspects of the program did you find enjoyable?
Question  3: what aspects of the program did you not like or thought could be improved?
Question  4: what were some of the changes (emotional, physical, etc.), if any, you noticed about yourself during or after the CROP service?
Question  5: what types of community-based activities, if any, do you think you may pursue after participating in the program that you were not pursuing before attending?
Question  6: overall, how satisfied were you with the program?
